# Damping and Stiffness Analysis of Sandwich Beam with 3D-Printed Honeycomb Core Filled with Magnetorheological Elastomer (MRE): An Experimental Approach

**DOI:** 10.3390/polym15183807

**Published:** 2023-09-18

**Authors:** Umer Sharif, Xinmei Xiang, Miaochang Zhu, Jun Deng, Jing Sun, Dauda Sh. Ibrahim, Orelaja Oluseyi Adewale

**Affiliations:** 1 School of Civil Engineering, Guangzhou University, Guangzhou 510006, China; umer.sharif@gzhu.edu.cn (U.S.); zhumiaochang@gzhu.edu.cn (M.Z.); dengjun@gzhu.edu.cn (J.D.); 2 State Key Laboratory of Mechanics and Control of Mechanical Structures, University of Aeronautics and Astronautics, Nanjing 210016, China; sidauda.mct@buk.edu.ng; 3 Department of Mechanical Engineering, Moshood Abiola Polytechnic Ogun State, Abeokuta 110252, Nigeria; 233179946@seu.edu.cn

**Keywords:** sandwich beam, magnetorheological elastomer, damping, damping coefficient, stiffness

## Abstract

The current study focuses on the production and experimental examination of sandwich beams consisting of an aluminum face sheet and 3D-printed honeycomb cores that are filled with magnetorheological elastomer (MRE). These cores are loaded with different ratios of (75/25)% and (50/50)% elastomer and magnetic particles, measured by weight. In order to ascertain the dynamic characteristics of sandwich beams, the constructed specimens were subjected to classic shock (free vibration) experiments, and these experiments were conducted under two conditions: with and without the application of a changing magnetic field at the free end and center of the beam. The results of the experiments suggest that the attenuation of the damping ratio exhibited satisfactory performance, particularly with respect to the structures that were being examined. The sandwich beam constructions proposed exhibited the ability to alter the damping ratio, damping coefficient, and stiffness through the application of a magnetic field. Nevertheless, an escalation in the applied magnetic field resulted in a reduction in stiffness values, while the values of the damping ratio and damping coefficient increased. Furthermore, significant variations in damping were observed when the magnets were located in the central regions of the structures.

## 1. Introduction

Magnetorheological elastomers (MREs) are a form of smart material with rheological properties that may be controlled in the presence of external magnetic fields [1]. MRE is a magneto-sensitive elastomer that consists of a rubber or elastomer matrix. The material’s composition comprises polarized magnetic particles, a rubber matrix consisting of either natural or synthetic components, and additives such as silicon oil [2]. MREs are composed of magnetic particles that are on a micron scale and are placed within a solid or gel-like matrix that lacks magnetic properties. This design choice effectively tackles issues commonly seen in magnetic resonance fluids (MRFs), such as particle mismatch and diminished strength of the magnetic resonance effect. The distribution of magnetic particles in MREs can display either isotropic or anisotropic characteristics, contingent upon the curing procedure involving the presence or absence of a magnetic field [3].

There are numerous analogous mechanical behaviors; however, the pre-yield area of the field-dependent modulus of MRE materials makes it one of their unique mechanical characteristics. Research [4] indicates that the highest increase in the MRE modulus is 40% more than the preliminary modulus, which is close to 0.6 MPa. Nevertheless, using MRE has many benefits over using other MR materials, such as the ability to operate in multiple degrees of freedom, variability in stiffness and frequency, and the elimination of the requirement to reposition magnetic particles in the presence of a magnetic field [5]. The polymeric or rubber matrix contains trapped magnetic particles that can be externally energized. Because of this, the particles are limited to moving in directions that are not influenced by a magnetic field. Nevertheless, the magnetic field causes a distortion in the magneto-active behavior of MREs when applied to soft elastic materials. The distinguishing characteristics of the MRE operational mode differentiate it from MR materials. The operating modes that are applicable to both isotropic and anisotropic MREs can be categorized into three main modes: shear mode, squeeze mode, and field-active mode. Nevertheless, it should be noted that MREs possess the capacity to undergo alterations in their physical configuration while operation in a field-active state or under the influence of a magnetic field, which is commonly referred to as magnetostriction [6].

The modeling of structural vibration response is of utmost importance in the process of sizing and designing structures. The precise estimation of natural frequencies holds significant importance in advanced domains such as civil engineering for seismic control [7,8], space exploration, and aerospace engineering, primarily due to the inherent unpredictability associated with these systems. The application of technology in enhancing the design and construction of structures results in a rise in mechanical stiffness and damping [9]. The presence of the MRE layer has an impact on the nonlinear dynamic characteristics of the system in the vicinity of fundamental and primary resonances. This influence leads to both qualitative and quantitative alterations in the manifestation or absence of a magnetic field [10].

Sandwich composite structures are frequently employed in various applications that require optimal ratios of stiffness to weight, strength to weight, and energy absorption capacity. These applications are typically required in the aerospace, automotive, civil, and naval industries [11,12]. The composition entails a low-density central component that is affixed between two slender outer layers. The core material and topology of sandwich composite structures significantly affect their mechanical behavior. Conventional honeycomb cellular cores exhibit superior stiffness, mechanical stability, and energy absorption compared to random porous foams when subjected to out-of-plane crushing [13,14]. MRE studies find the sandwich panel’s rigidity to be customizable through the cell’s geometric layout, making it an attractive construction option.

Cellular structures with varying shapes and sizes can be utilized to regulate the mechanical behavior of the honeycomb through manufacturing techniques such as 3D printing [15]. Sandwich composite structures and MREs typically involve bonding silicone-based MREs to metallic skins. Sandwich panel structures necessitate a design with high compressive and shear strength, emphasizing the significance of the core bonding to the face material, which substantially affect the stiffness, strength, and durability of the structures [16].

Prior research has explored the use of MREs in various applications such as ATVAs [17,18,19,20,21,22], vibration isolators [23,24,25], adaptive base isolators [26,27], and stiffness-tunable suspensions and mounts [28]. Furthermore, scholarly research has been conducted on the subject of material empirical modeling, as evidenced by studies [29,30,31,32,33], Additionally, investigations have been carried out on material development and property testing [34,35,36], innovative device design and characterization [37,38], performance evaluation [39,40,41,42], and sandwich beams [43,44]. Several studies have also investigated the dynamic analysis of sandwich constructions made with magnetorheological gel (MRG) MREs [45,46,47,48]. The sandwich construction exhibits a notable ratio of stiffness to weight, which enables the effective integration and optimization of diverse materials for both the surface and core layers. The sandwich structure of the MRE demonstrates the ability to attain adjustable levels of stiffness and damping, as well as rigidity and resistance to deformation. This design takes into account the mitigation of vibration and noise.

The significance of this approach lies in the utilization of a hexagonal honeycomb core material constructed of nylon and Resin8000, which is 3D-printed and filled with MRE, while featuring aluminum face sheets. This methodology facilitates the establishment of stronger connections between the central core and outside skins, resulting in enhanced mechanical properties of the sandwich composite structure. The main objective of this work is to determine the structural viability of a hexagonal honeycomb cored sandwich panel reinforced using MREs. Furthermore, the objective of this research is to advance our understanding of the mechanical and dynamic properties such as the damping coefficient, damping ratio, and stiffness of sandwich beam constructions that integrate a hexagonal honeycomb core fabricated using 3D printing that consists of a unique material and is filled with different ratios of MREs. There is currently a lack of prior research examining the damping and stiffness characteristics of proposed structures under different magnetic fields applied at various locations. To demonstrate the possibility of achieving customizable levels of stiffness and damping, as well as rigidity and resistance to deformation, the goal of this study was to investigate the impact of magnetic fields on the dynamic properties of several suggested structures at different locations.

## 2. Sandwich Beam Structure Design and Fabrication

The primary objective of this study is to investigate the dynamic behavior of magnetorheological composite beams. This section will present a detailed account of the manufacturing procedure utilized for the sandwich beam structures proposed in this research. The report will be divided into three sections, which will encompass a comprehensive explanation of the honeycomb core manufacturing process, the preparation of the magnetorheological elastomer (MRE), and the assembly of the proposed sandwich beam structures.

### 2.1. Honeycomb Core Design and Manufacturing

**Figure 1:**
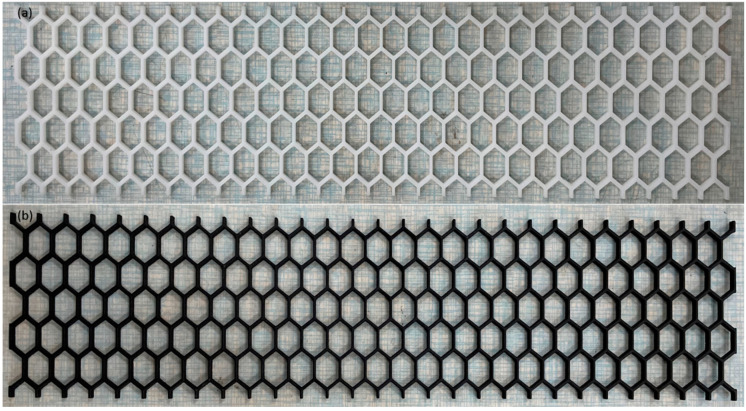
Proposed honeycomb cores: (**a**) Resin 8000, (**b**) Nylon.

Initially, the honeycomb core was created and subsequently filled with MREs in different proportions. The honeycomb core was designed using SolidWorks 14, a CAD tool developed by Dassault System. This study employed the conventional hexagonal honeycomb configuration. WeNext, a Chinese company, 3D-printed the honeycomb cores. Stereolithography (SLA) was used for polymerization with a resolution of 0.1 mm. Two honeycomb materials, Nylon and Resin 8000, were selected for comparison. The materials were selected based on their 3D-printability, accessibility, and mechanical characteristics. The proposed sandwich beam structure employs Resin 8000 and Nylon honeycomb structures, as depicted in Figure 1.

### 2.2. Magnetorheological Elastomer (MRE) Core Development

**Figure 2:**
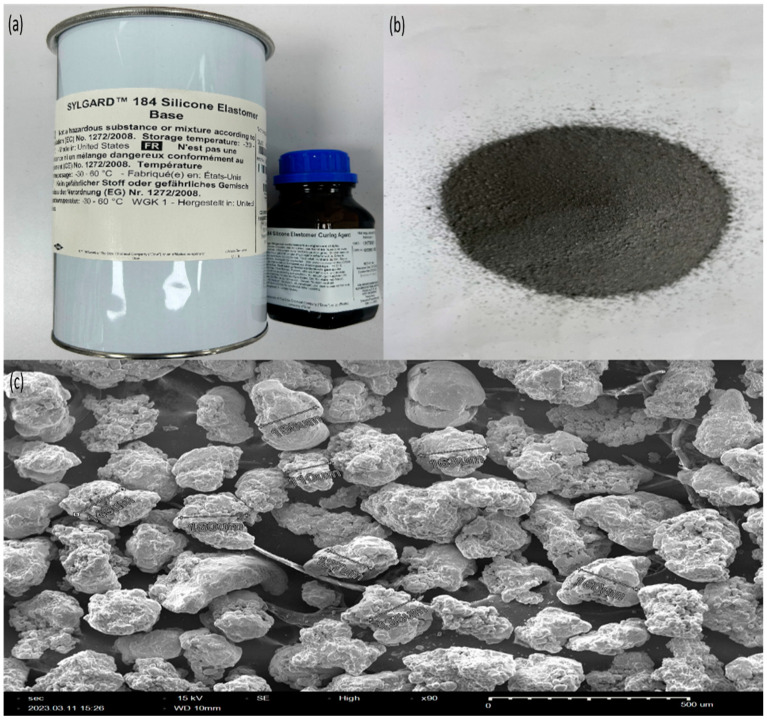
Materials used for manufacturing of MRE. (**a**) Silicon elastomer kit and (**b**) magnetic particles. (**c**) SEM image of magnetic particles.

**Figure 3:**
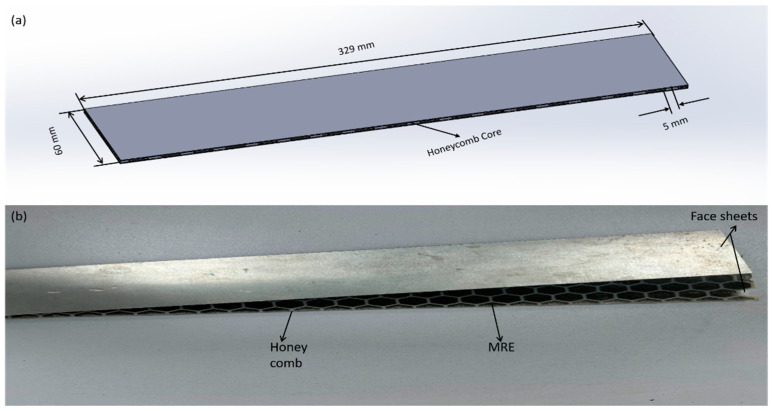
(**a**) Proposed sandwich beam. (**b**) Composition of sandwich beam.

The magnetorheological core material was produced by mixing magnetic powder from CMS Magnetics, China, with a size distribution of 110 µm to 170 µm (SEM image in Figure 2c) with uncured silicone elastomer from SYLGARD, city Germany, in weight ratios of 25% and 50%. A stirrer was utilized for the purpose of blending and achieving homogeneity of the materials. The MR material was cured using a Sylgard curing agent from Germany. Figure 2 illustrates the MRE manufacturing materials. The MRE was poured into the honeycomb and was cured at room temperature. After the completion of this phase, distinct molds were employed to ensure the formation of identical shapes for the 25% and 50% blends. The material underwent a 24-h isotropic curing process without magnetic influence. Figure 3 illustrates the MRE filled with honeycomb core. The purpose of utilizing the silicon elastomer for manufacturing the proposed MRE is its unique properties like stability and good adhesion with metals.

### 2.3. Fabrication of Magnetorheological Sandwich Beams

This study utilized test specimens produced after creating the MRE core and honeycomb structure. The honeycomb structure was filled with MRE material and enclosed by two 1 mm thick aluminum sheets measuring 60 mm in width and 329 mm in length, provided by We Next in China (Shenzhen). The two layers were assembled with a 0° orientation and fused together using DOW-supplied epoxy resin to form the aluminum/MRE honeycomb core, as depicted in Figure 3. Aluminum exhibits superior mechanical vibration characteristics owing to its low damping properties and high rigidity in comparison to MR elastomers. Aluminum has a relative magnetic permeability of zero, implying that it does not impact the magnetic field’s distribution or intensity [7,44]. Table 1 displays the composition and dimensions of the sandwich beam structures examined in the present investigation.

## 3. Design of Experiment

**Figure 4:**
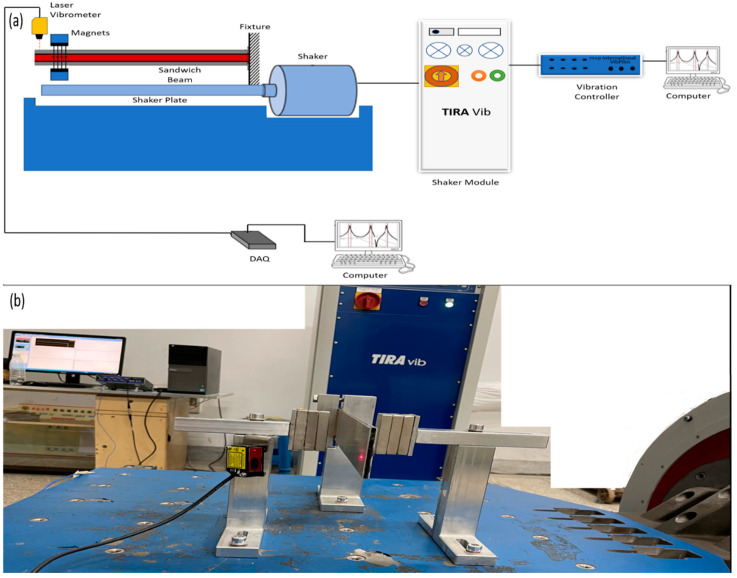
(**a**) Schematic diagram of experimental setup. (**b**) Experimental setup.

The acquisition of the results necessitated the implementation of a well-designed experimental configuration. The experimental configuration comprised two distinct portions. The initial phase of the experiment entailed performing a conventional shock test, wherein magnets were strategically placed at the unfettered extremity to examine and delineate the modal response of the structures under investigation. Following this, a series of classic shock tests were performed on the structure by strategically positioning magnets at its central point in order to stimulate unrestricted oscillation. The shaker exerted an external force. The schematic representation of the experimental setup is depicted in Figure 4a.

This study employed honeycomb sandwich cantilevered beams with MRE filling, which had dimensions of 300 mm in length, 60 mm in breadth, and 5 mm in thickness. The experimental setup depicted in Figure 4b consists of magnets, a laser vibrometer, a shaker, and data gathering equipment. The experiment employed the VibPilot vibration controller and VibRunner software, both manufactured by m+p International, as well as a TIRA Vib Shaker provided by TIRA Gmbh, Germany. The signals were obtained using a National Instruments model no. 9432 Data Acquisition (DAQ) board and a Panasonic model no. HG-C1100 laser vibrometer. A laser vibrometer is preferred over an accelerometer due to its non-invasive nature, as it does not alter the system’s mass or, consequently, the vibration outcomes. The fundamental frequency of the structures was determined through a classic shock test during the free vibration analysis. The obtained signals were analyzed using the SO Analyzer software. To account for the sandwich beam’s 10 mm free end, Nd-Fe-B permanent magnets with a performance grade of N38 and dimensions of 60 mm × 30 mm × 10 mm were placed 20 mm from the free end and at the center of the structures.

## 4. Results and Discussion

### 4.1. Influence on Damping Ratio

**Figure 5:**
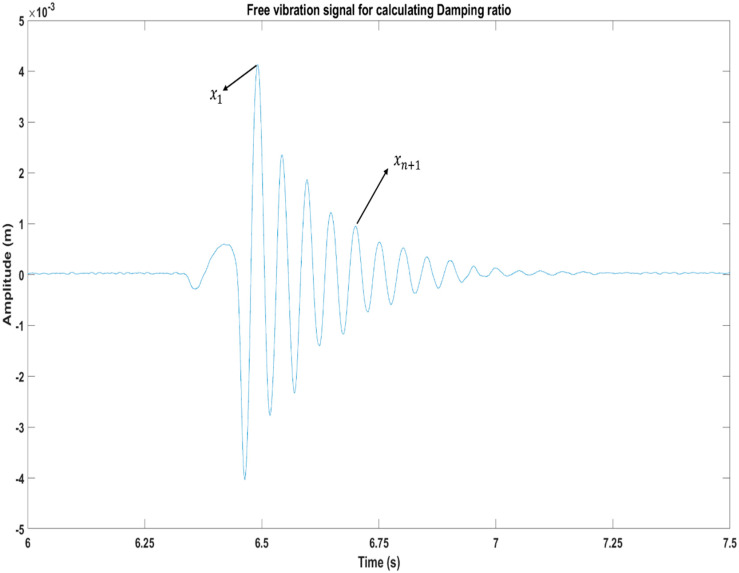
Amplitude–time signal for calculating damping ratio.

Accurate estimation of structural damping is crucial for dynamic analysis involving mechanical vibrations. Shock tests were performed on various structures using different magnet intensities ranging from 0 G to 7.5 × 10^3^ G, as shown in Figure 5, to analyze the damping ratio. Tests were performed at a 5g impact acceleration generated by a shaker. The structure’s signal was subsequently noted and analyzed using a laser vibrometer, DAQ, and SO analyzer. The presence of silicone prevents ferromagnetic particles from acquiring a preferred orientation, resulting in minimal changes in structural damping. To enhance damping while adjusting the magnetic field, a viable approach is to incorporate a less rigid face material in the beam structure. Because of this, a substantial change in the investigated system’s structural damping can be observed. The beam’s structural damping has been thoroughly analyzed in this work. Equations (1) and (2) were used to the temporal signal shown in Figure 5 to determine the logarithmic decrement and damping ratio of the proposed structures when exposed to varying magnetic intensities.

(1) $$Logarithmic\;decrement=S=\frac{1}{n}\ln\left(\frac{x_{1}}{x_{n+1}}\right)$$

(2) $$Damping\;ratio=\delta =\frac{S}{2\pi }$$

Several parameters, apart from the applied magnetic field, have an impact on the damping characteristics of sandwich constructions based on MREs. The elements encompassed in this analysis are the characteristics of the face layers in terms of their material composition and thickness, the thickness of the core layer, the boundary conditions imposed on the system, external disturbances acting upon it, the various modes of vibration shown, interfacial slip between particles and the matrix, and the influence of temperature. The absence of this behavior in the proposed structures may be attributed to the absence of a linear relationship between the deviation of the damping ratio and the applied magnetic field.

#### 4.1.1. Magnets at Free End

In general, raising the applied magnetic field at the free end from 0 to 2.5 × 10^3^ G led to an increase in the damping ratio. However, further increasing it to 7.5 × 10^3^ G resulted in a decrease in the damping ratio. The sandwich beam constructions, composed of Resin8000 and nylon honeycomb cores, filled with a mixture of MRE (50/50)% exhibited a significant difference in the maximum damping ratio of 33.33% and 42% when exposed to a magnetic field intensity of 7.5 × 10^3^ G. The sandwich beam configuration with a Resin8000 honeycomb core and incorporating equivalent quantities of MRE demonstrated a linear response. The maximum deviation observed is 33.33% at a magnitude of 7.5 × 10^3^ G. The sandwich beam structure that incorporates a Nylon honeycomb core and contains a (50/50)% mixture of MRE demonstrated enhanced performance compared to other structures. It exhibited a maximum deviation at 7.5 × 10^3^ G.

#### 4.1.2. Magnets at Center

The experimental investigation involved the testing of sandwich beam structures composed of Resin8000 matrix material filled with two different ratios of MRE, namely (75/25)% and (50/50)%. A magnetic field with a magnitude of 7.5 × 10^3^ G was applied at the center. The observed maximum fluctuations in the damping ratio were determined to be 58.28% and 51.28% for the respective cases. With a maximum deviation of 51.26% at a magnitude of 7.5 × 10^3^ G, the sandwich beam construction with a Resin8000 honeycomb core and (50/50)% MRE displayed linear properties. In contrast, under 7.5 × 10^3^ G, the central point of a sandwich beam arrangement with a Nylon honeycomb core and a (75/25)% MRE composition deviated by only 14.54%.

**Figure 6:**
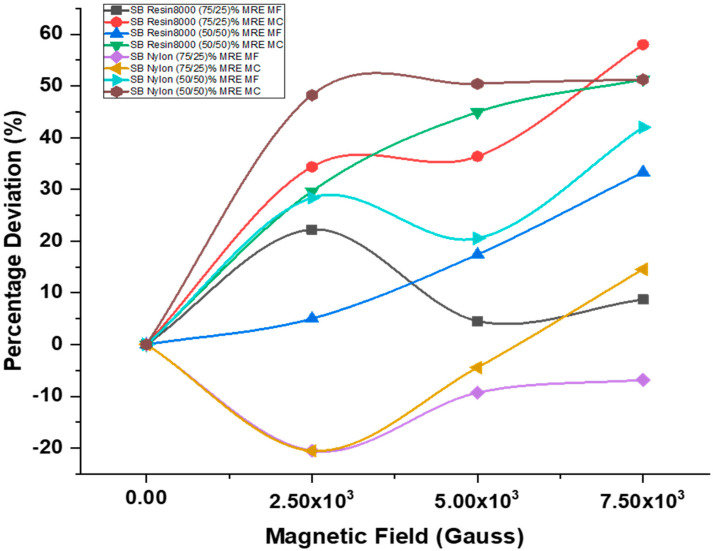
Magnetic intensity influence on damping ratio.

For a variety of proposed structures and magnetic field intensities, Figure 6 shows the percentage variation in the damping ratio as a function of the applied magnetic field. All the sandwich beam structures we looked at with varied magnetic intensities are included in Table 2 with their calculated damping ratio values.

### 4.2. Influence on Damping Coefficient

The mechanical and rheological properties of an elastomeric material containing magnetizable particles can be reversibly modified by an external magnetic field. The aforementioned phenomenon is commonly known as the magnetorheological effect. The field dependency of magnetorheological elastomer (MRE) properties can be attributed to the existence of field-induced dipole magnetic forces among the particles. The mechanical behavior of MR elastomers is influenced by the presence of magnetizable particles incorporated within the matrix.

A smart material is one that can be controlled by manipulating the magnetic field. Thus, a damping coefficient for MRE is needed in order to optimize the application in the attribute. Damping is a unique characteristic that has an effect on the mechanical system’s vibration. This property has the effect of reducing the amplitudes of vibrations by dissipating the energy stored during the vibratory movement. The energy loss is the primary characteristic that is determined by the damping coefficient. As a result, the damping characteristics of the proposed sandwich beam structures were thoroughly investigated under various magnetic field configurations. The damping coefficient of all the structures under investigation discussed in the above sections was calculated using Equation (3).

(3) $$\text{Equivalent Damping coefficient}=C_{eq}=\frac{M_{eq}w_{n}}{Q}$$

where “$M_{eq}$” is the equivalent mass of the beam under investigation and can be calculated by using Equation (4).

(4) $$M_{eq}=\frac{33}{140}M_{b}$$

“$M_{b}$” represents the mass of the beams and is presented in Table 1.

(5) $$w_{n}=2\pi f$$

“$w_{n}$” is the angular natural frequency and “f” is the natural frequency of the proposed structures presented in Table 1 and “δ” represents the damping ratio presented in Table 2.

(6) $$Q=\frac{1}{2\delta }$$

#### 4.2.1. Magnets at Free End

The behavior observed for the damping response was similar to the damping ratio as presented in Section 4.1. When the applied magnetic field at the free end was increased from 0 to 2.5 × 10^3^ G, in most cases, the damping ratio increased, except when the magnetic field was raised to 7.5 × 10^3^ G, where it decreased. The damping coefficient changed significantly in sandwich beam structures made of Resin8000 (75/25)% MRE and Nylon honeycomb cores filled with (50/50)% MRE. The Resin8000 sandwich beam structure changed 16.62% and the Nylon honeycomb core sandwich beam structure 19.77%. The free end of the structure changed when a 2.5 × 10^3^ G magnetic field was applied. The sandwich beam structure with a Resin8000 honeycomb core filled with (50/50)% MRE was linear, with a maximum variation of 12.20% at 7.5 × 10^3^ G. However, the Nylon honeycomb core and 75% MRE combination showed a declining trend, peaking at −12.94% under 7.5 × 10^3^ G magnetic field intensity.

#### 4.2.2. Magnets at Center

More deviation in the damping coefficient was observed when the magnets were placed at the center of the structures, and this phenomenon was seen in all the cases. The sandwich beam structures consisting of Resin8000 filled with a mixture of (75/25)% magnetorheological elastomer (MRE) exhibited a maximum deviation in the damping coefficient of 57.89% when a magnetic field of 7.5 × 10^3^ G was applied. In contrast, the sandwich beam with a Nylon honeycomb core and a composite consisting of (50/50)% MRE exhibited the most significant variation in the damping coefficient, amounting to 43.03%, upon the application of a magnetic field of 2.5 × 10^3^ G in its central region. The sandwich beam structures, one including a Resin8000 honeycomb core and the other incorporating a Nylon honeycomb core with a (75/25)% MRE, exhibited varying degrees of linear response when exposed to a 7.5 × 10^3^ G force. The Resin8000 honeycomb structure exhibited a maximum deviation of 57.89% and 39.78%, while the Nylon honeycomb structure with (75/25)% MRE showed a minimum deviation of 6.57% when the force was applied at the center of the structure.

**Figure 7:**
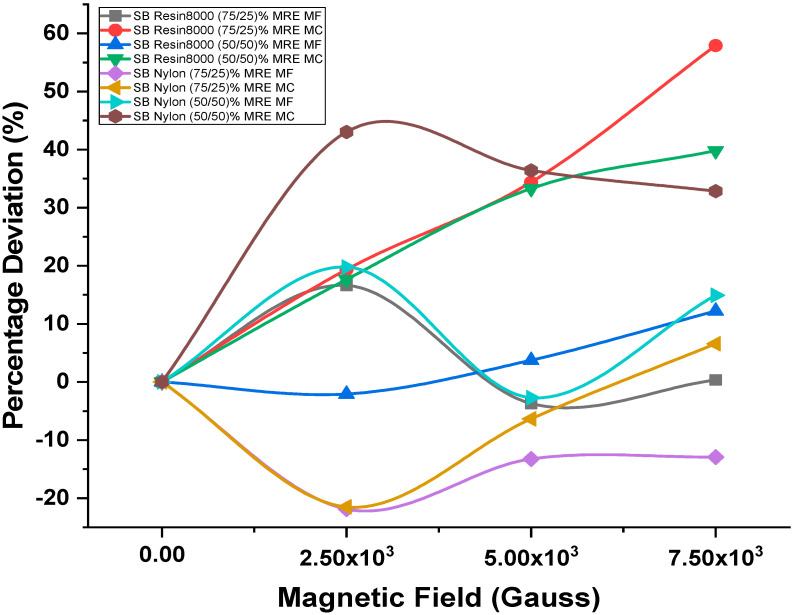
Magnetic intensity influence on damping coefficient.

Figure 7 illustrates the percentage variation observed in the damping coefficient as a function of the applied magnetic field for several proposed structures, encompassing a range of applied magnetic fields. Furthermore, Table 3 presents a comprehensive overview of the computed damping coefficient values for the various sandwich beam architectures that have been examined, taking into account the varying magnetic intensities.

### 4.3. Influence on Stiffness

The polarization of magnetic particles occurs when they are exposed to a magnetic field. In this particular scenario, the particles mutually apply forces to each other, leading to an increase in the rigidity of the substance. Upon the removal of the magnetic field, the magnetorheological elastomer (MRE) returns to its initial state. Hence, with the utilization of a magnetic field, it is possible to manipulate the mechanical characteristics of magnetorheological elastomers (MREs), including the storage and loss modulus. In contrast, sandwich structures that incorporate MREs function within the pre-yield regime and are characterized by their modulus that varies with the applied field. The utilization of MRE materials in structures allows for the manipulation of the natural frequency, which is contingent upon the equivalent stiffness of the structure. This is made possible by the materials’ capacity to modify their modulus in response to external stimuli. Variations in the shear modulus result in a displacement of the stiffness parameters. The prevention of resonance or other linked phenomena inside a system can be achieved by the adjustment of stiffness. For this purpose, the stiffness was calculated for all the proposed structures with a varying field applied at the free end and center of the structures by using Equation (7).

(7) $$K_{eq}=w_{n}^{2}\times M_{eq}$$

#### 4.3.1. Magnets at Free End

Because of the linearity of the stiffness response seen, it can be concluded that the stiffness value decreases as the intensity of the applied magnetic field increases. The sandwich beam structure with a Resin 8000 honeycomb core and (75/25)% MRE had a stiffness of 1062.53 N/m when tested without a magnetic field applied. When the applied magnetic field reached 7.5 × 10^3^ G, this number decreased to 887.47 N/m, representing a deviance of 16.47%. For the identical structure with (50/50)% MRE, the value reduced from 572.86 N/m to 331.41 N/m, a 42.41% reduction in the value. Using sandwich beam constructions composed of nylon honeycomb cores with (75/25)% MRE, a deviation of 10.69% was detected, and the stiffness value was reduced from 359 N/m to 321.32 N/m. In contrast, when the applied magnetic field hit its limit of 7.5 × 10^3^ G, the structure with Nylon honeycomb cores filled with (50/50)% MRE displayed a maximum change in stiffness of 53.54%, reducing the value from 314.41 N/m to 146.08 N/m.

#### 4.3.2. Magnets at Center

**Figure 8:**
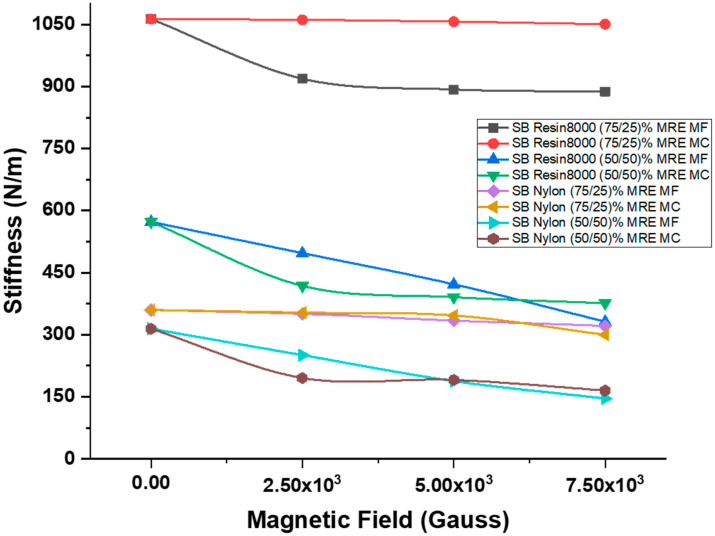
Magnetic field intensity influence on stiffness.

Similarly to when magnets were placed at the free end of the structure, when magnets were placed in the center of the structure, the response was also linear, but there was a smaller deviation in the stiffness response. When tested without a magnetic field, the sandwich beam structure with a Resin 8000 honeycomb core and (75/25)% MRE had a stiffness of 1062.53 N/m. Upon reaching a magnetic field strength of 7.5 × 10^3^ G, this number dropped to 1050.14 N/m, which represents a deviation of 1.1%. The value for the identical structure with (50/50)% MRE was reduced from 572.86 N/m to 375.86 N/m, a reduction of 34.39%, which is lower than the values obtained when magnets were placed at the free ends and the same magnetic field was applied. A deviation of 16.67% was discovered in sandwich beam constructions composed of Nylon honeycomb cores with (75/25)% MRE, and the stiffness value was reduced from 359 N/m to 299.83 N/m when using Nylon honeycomb cores with MRE. When the applied magnetic field reached its maximum strength, the structure with Nylon honeycomb cores filled with (50/50)% MRE showed the greatest change in stiffness, with a maximum change of 47.47%, resulting in a reduction in value from 314.41 N/m to 165.17 N/m. Figure 8 illustrates the percentage deviation in stiffness relative to the applied magnetic field for different proposed structures at varying levels of magnetic field intensity. Additionally, Table 4 provides a summary of the stiffness values computed for all sandwich beam structures examined under the influence of different magnetic intensities.

## 5. Conclusions and Future Work

The sandwich beams, which were constructed using aluminum face sheets and a hexagonal honeycomb core manufactured via 3D printing, were exposed to classic shock experiments. The honeycomb core was filled with magnetorheological elastomer at various proportions. The dynamic properties of the sandwich beams were analyzed using magnetic fields ranging from 0 to 7.5 × 10^3^ G. The experimental findings demonstrated that magnetic fields have a beneficial influence on both the damping ratio and coefficient of the adaptive beam made of magnetorheological elastomers (MREs). The alteration in magnetic field intensity has the potential to modify the damping characteristics. The damping ratio of sandwich constructions with (50/50)% MRE of Nylon and Resin honeycomb cored sandwich beams was increased by 51.26% and 51.28%, respectively, when 7.5 × 10^3^ G was applied at the center of the structure. The damping coefficient of the structures containing (50/50)% MRE followed the same pattern.

When exposed to forced vibration, all specimens exhibited a reduction in stiffness as the intensity of the magnetic field increased. However, the structure containing a Nylon honeycomb core and a (50/50)% ratio of MRE demonstrated the most notable deviation. Specifically, this structure displayed a deviation of approximately 53.54% and experienced a decrease of 47.47% when the maximum magnetic field was applied at both the free end and center of the structures, respectively. Magnetization has been shown to be more successful at modifying specimens’ damping and stiffness properties with (50/50)% MRE when compared to the structures with (75/25)% MRE used in this study.

## Figures and Tables

**Table 1:** Composition and dimensions of sandwich beams.

Sandwich Beam Samples	Composition																Sandwich Beam			
	Face Sheets					Honeycomb						Honeycomb with MRE								
	Material	Dimensions			Mass (g)	Material	Dimensions				Mass (g)	Material	Dimensions			Mass (g)	Dimensions			Mass (g)
		L (mm)	W (mm)	T (mm)			L (mm)	W (mm)	T (mm)	S (mm)			L (mm)	W (mm)	T (mm)		L (mm)	W (mm)	T (mm)	
1	Aluminum	329	60	1	51.90	Resin 8000	329	60	3	1.2	15.90	75% Silicon, 25% Magnetic particles	329	60	3	95.80	329	60	5	199.60
2						Resin 8000						50% Silicon, 50% Magnetic particles				143.30				247.10
3						Nylon					10.80	75% Silicon, 25% Magnetic particles				93.40				197.20
4						Nylon						50% Silicon, 50% Magnetic particles				138.60				242.40

**Table 2:** Damping ratio behavior of sandwich beam structures.

Sandwich Beam Sample	Magnetic Field (Gauss)	Damping Ratio (MF)	Deviation (%)	Damping Ratio (MC)	Deviation (%)
Resin8000 (75/25)% MRE	0	0.0021	0%	0.0021	0%
	2.5 × 10^3^	0.0027	22.22%	0.0032	34.37%
	5.0 × 10^3^	0.0022	4.54%	0.0033	36.37%
	7.5 × 10^3^	0.0023	8.69%	0.0050	58%
Resin8000 (50/50)% MRE	0	0.0038	0%	0.0038	0%
	2.5 × 10^3^	0.0040	5%	0.0054	29.63%
	5.0 × 10^3^	0.0046	17.39%	0.0069	44.92%
	7.5 × 10^3^	0.0057	33.33%	0.0078	51.28%
Nylon (75/25)% MRE	0	0.0047	0%	0.0047	0%
	2.5 × 10^3^	0.0039	−20.51%	0.0039	−20.51%
	5.0 × 10^3^	0.0043	−9.30%	0.0045	−4.44%
	7.5 × 10^3^	0.0044	−6.82%	0.0055	14.54%
Nylon (50/50)% MRE	0	0.0058	0%	0.0058	0%
	2.5 × 10^3^	0.0081	28.39%	0.0112	48.21%
	5.0 × 10^3^	0.0073	20.54%	0.0117	50.42%
	7.5 × 10^3^	0.0100	42%	0.0119	51.26%

**Table 3:** Damping coefficient behavior of sandwich beam structures.

Sandwich Beam Sample	Magnetic Field (Gauss)	Damping MF (Ns/m)	Deviation (%)	Damping MC (Ns/m)	Deviation (%)
Resin8000 (75/25)% MRE	0	0.0296	0%	0.0296	0%
	2.5 × 10^3^	0.0355	16.62%	0.0367	19.34%
	5.0 × 10^3^	0.0285	−3.71%	0.0451	34.36%
	7.5 × 10^3^	0.0297	0.33%	0.0703	57.89%
Resin8000 (50/50)% MRE	0	0.0439	0%	0.0439	0%
	2.5 × 10^3^	0.0430	−2.09%	0.0533	17.63%
	5.0 × 10^3^	0.0456	3.72%	0.0658	33.28%
	7.5 × 10^3^	0.0500	12.20%	0.0729	39.78%
Nylon (75/25)% MRE	0	0.0384	0%	0.0384	0%
	2.5 × 10^3^	0.0315	−21.90%	0.0316	−21.52%
	5.0 × 10^3^	0.0339	−13.27%	0.0361	−6.37%
	7.5 × 10^3^	0.0340	−12.94%	0.0411	6.57%
Nylon (50/50)% MRE	0	0.0491	0%	0.0491	0%
	2.5 × 10^3^	0.0612	19.77%	0.0862	43.03%
	5.0 × 10^3^	0.0478	−2.72%	0.0772	36.39%
	7.5 × 10^3^	0.0577	14.90%	0.0731	32.83%

**Table 4:** Stiffness behavior of sandwich beam structures.

Sandwich Beam Sample	Magnetic Field (Gauss)	Stiffness MF (N/m)	Deviation (%)	Stiffness MC (N/m)	Deviation (%)
Resin8000 (75/25)% MRE	0	1062.53	0%	1062.53	0%
	2.5 × 10^3^	918.58	13.55%	1060.76	0.17%
	5.0 × 10^3^	892.35	16.02%	1056.33	0.58%
	7.5 × 10^3^	887.47	16.47%	1050.14	1.1%
Resin8000 (50/50)% MRE	0	572.86	0%	572.86	0%
	2.5 × 10^3^	497.17	13.21%	418.12	27.01%
	5.0 × 10^3^	421.85	26.36%	390.69	31.80%
	7.5 × 10^3^	331.41	42.41%	375.86	34.39%
Nylon (75/25)% MRE	0	359.81	0%	359.81	0%
	2.5 × 10^3^	350.11	2.69%	353.15	1.85%
	5.0 × 10^3^	334.07	7.15%	346.57	3.68%
	7.5 × 10^3^	321.32	10.69%	299.83	16.67%
Nylon (50/50)% MRE	0	314.41	0%	314.41	0%
	2.5 × 10^3^	250.42	20.35%	195.34	37.87%
	5.0 × 10^3^	187.90	40.24%	190.79	39.32%
	7.5 × 10^3^	146.08	53.54%	165.17	47.47%

## Data Availability

Data can be provided upon request.
